# Mr. W. White on Strictures of the Rectum, and Other Affections, Which Diminish the Capacity of That Intestine; with Cases and Engravings

**Published:** 1820-09-01

**Authors:** 


*Observations on Strictures of the Rectum, and other Affections which diminish the Capacity of that Intestine, including Spasmodic Constriction of the Anus, the Hæmorrhoidal Tumours, Excrescences, and Prolapsus Ani: and the Mode of Treatment, accompanied with Cases and Engravings.* By W. White, Member of the Royal College of Surgeons, London, and one of the Surgeons to the City of Bath Infirmary and Dispensary. Third Edition improved. One vol. 8vo. pp. 172. 1820.

Those diseases affecting the channels by which excrementitious matters are carried off from the body, are scarcely less

---

terrible in their consequences, though more slow in their progress, than the diseases incidental to organs of supply, and what are termed the vital viscera. Who would not prefer a chronic inflammation of the lungs to a similar disease in the bladder? Is not an ulcer in the rectum almost as much to be dreaded as an abscess in the liver? It is now well ascertained, that all the muscular and membranous tubes of the human body, as the œsophagus, stomach, small intestines, colon, rectum, ureter, and urethra, are liable to spasmodic and organic constrictions, which often render life miserable, and not seldom terminate our existence. Strictures of the rectum have not excited much attention till of late years. Dr. Sherwin, who is the first in this country, who gave a complete history of the symptoms attending contracted rectum arising from scirrhus, has the following melancholy prognosis.

"The disease comes on in the most gradual and imperceptible manner; slow in its progress, but terrible in its consequences; it yields not to medical assistance, but must, under the best management, become ultimately fatal."

This prediction, Mr. White thinks, is true, as it respects the scirrho-contracted rectum; but not so with regard to simple stricture. Simple stricture of the rectum, our author considers as of frequent occurrence, but not generally *within the reach of the finger*, as some eminent practitioners believe. A scirrhous state of the rectum, on the other hand, is commonly within reach of the finger.

Mr. White does not wonder at strictures of the rectum being frequently overlooked, since, like strictures of the urethra, they are capable of exciting morbid action in distant parts of the system. The symptoms of their early stages too, are apt to be confounded with "habitual costiveness, piles, stomach complaints, or bilious obstructions;" while those attendant on the more advanced progress of the malady, are attributed to chronic diarrhœa.

"What Dr. Sherwin has so justly observed with regard to scirrhus of the rectum, is likewise strictly applicable to simple stricture. 'There is no disease (he says) to which the human frame is incident, that is more liable to be misunderstood. Diarrhœa, dysentery, tenesmus, cholic, painful distension of the abdomen, inflammation of the bowels, and iliac passion, which are each of them formidable, and often fatal in themselves, may be succesive symptoms of the scirrhous rectum. Under some one of these appearances, it is highly presumable, that many patients have died without the real cause having ever been assigned or suspected; and even when it is suspected, and becomes an object of manual investigation, may be easily mistaken for an enlargement of the prostate gland or scirrhous uterus." 8.

Though no age is entirely exempt from this disease, yet it does not often appear before the meridian of life.

The most simple form, according to our author, of constriction in the lower part of the intestinal canal, is that produced by spasm, which is an inordinate degree of contraction in the muscular coat of the intestine, from some irritation. Numerous repetitions, or long continuance of this spasmodic, may produce permanent contraction.

"Whenever, therefore, a spasmodic constriction at the sphincter is discovered, it becomes highly expedient to ascertain the state of the passage above, by introducing a bougie not less than ten or eleven inches in length, and of a diameter adapted to the degree of constriction at the sphincter; for if an obstruction should remain higher up the passage unexplored, any attempt to remove the constriction below will prove of little avail." 12.

Mr. White observes, that sometimes, when a stricture is within reach of the finger, it feels like a membranous ring, and where there is much pressure from an accumulation of fæces above, a regular contraction and dilatation of the muscular coat of the intestine may be felt. Although this stricture be permanent, yet our author thinks it may be properly termed spasmodic, as it is not attended by any sensible thickening or induration of the intestinal coats, but merely a contraction of their muscular fibres. In such cases, the gut above is very apt to become distended into a pouch, in consequence of reiterated accumulations; and this may lead a surgeon to suspect that he feels a tumour in the rectum, upon the first examination.

The next form of contraction which our author describes, is what he terms simple permanent stricture, which, he thinks, occurs more frequently than the spasmodic, and differs from the latter, in being attended with some slight alteration in the structure of the part where the stricture takes place, consisting of a thickening of the coats of the intestine, not by any means considerable, and, in general, only occupying a very small portion of the circumference of the gut.

"On passing the bougie slowly through the stricture, the muscular action may be distinctly discerned, which is not the case when the rectum is become thickened and indurated to any great extent, as commonly happens in a scirrhous state of the intestine." 17.

The cause of this coarctation, Mr. White thinks, with reason, to be a contraction and gradual thickening of the muscular coat of the gut, the inner membrane, though sometimes giving origin to a number of little processes that form a circle round the intestine, appearing incapable of contraction, of itself. This simple stricture of the rectum is sometimes attended with prolapsus ani, fleshy excrescences, and hæmorrhoidal tubercles.

"Not unfrequently, a contracted state of the rectum occurs as a consequence of the venereal disease. When the disorder proceeds from this cause, it generally commences with an appearance either of ulceration, or excrescence about the verge of the anus. The sphincter ani becomes gradually contracted, and the disease extending upwards within the rectum, a considerable thickening and induration of the coats of the intestine take place, which produce great irregularity and contraction in the passage. Sometimes there is a continued line of contraction from the anus, as far as the finger can reach, then terminating in a kind of cartilaginous border, the inner membrane having a thickened and condensed feel. There is often a discharge indicating a diseased, if not ulcerated state of the inner membrane above the contracted portion of intestine. All the cases which I have hitherto met with of this nature, have occurred in females, and they have uniformly proved incurable, when attended with the structural derangement just described." 18.

The rectum is also liable to contraction from tubercles situated immediately above the sphincter ani, very different from the soft, bluish, hæmorrhoidal tubercles, which often surround the anus. These last protrude when the patient strains; and when returned within the sphincter, no hardness can be perceived in the gut. It is the reverse with the other tubercles; they do not come below the sphincter, and they have an indurated feel.

Another cause of contraction, and that the most deplorable of all, is scirrhus. Many instances of this kind are recorded by authors; and as it is generally incurable, Mr. White thinks that other cases of contraction may have been mistaken for true scirrhus, and, on that account, abandoned.

"It is proper to notice, as one distinguishing mark of true scirrhus, that it generally commences not at the lower extremity of the rectum, as in the last-mentioned instances of contraction, arising from a venereal cause, but (as Dr. Baillie observes) two or three inches above the outer sphincter, and there is a sound capacious portion of the bowel between the stricture and this sphincter.* The scirrhus commonly surrounds, and sometimes occupies nearly the whole cavity of the rectum, from the extensive thickening and induration of its coats, particularly the muscular; and in the advanced stage of the disease, there is either an abrasion or entire destruction

* I do not mean to infer from this remark, that the disorder may not extend in its progress to the extremity of the gut.

of its internal membrane, attended by a serous, or thin sanious discharge. The severe sufferings of the patient during the progress of this dreadful malady, and its more rapid advance to a fatal termination, will also serve to distinguish it from other species of contraction, when it would be difficult sometimes to decide from mere local investigation.

"The following are the appearances discovered by dissection, which are so accurately described by Dr. Baillie, as to render any description of mine unnecessary. 'It (the scirrhus) sometimes extends over a considerable length of the gut, viz. several inches; but generally it is more circumscribed. The peritoneal, muscular, and internal coats are much thicker and harder than in a natural state. The muscular, too, is subdivided by membranous septa, and the internal coat is sometimes formed into hard irregular folds. It often happens, the surface of the inner membrane is ulcerated, producing cancer. Every vestige of the natural structure is occasionally lost, and the gut appears changed into a gristly substance'." 20, 21.

It is necessary to recollect, also, that the rectum is liable to have its capacity lessened by other affections of the neighbouring parts, especially a scirrhous uterus — sometimes an enlargement of the ovarium, or a diseased prostate.

*Etiology*. Although Mr. White does not deny that irritation or inflammation of the inner membrane of the gut may, in some instances, be the cause of stricture; yet, he thinks, that such instances are very few, compared with those of simple permanent stricture, where no such cause can possibly be assigned. He is inclined to think, that—

"The most frequent predisposing cause, is the gut being somewhat narrower about the termination of the sigmoid flexure of the colon, than it ought to be, for the purpose of allowing a free and easy passage to the fæces. I was led to this opinion in consequence of patients having so often stated to me, that so long as they could remember, they never had a natural motion, without experiencing more or less difficulty: it will then appear obvious, that if the passage should be preternaturally small, it must necessarily form an impediment to the free discharge of the fæces, and thus a foundation will be laid for a greater degree of contraction, as reiterated pressure from the accumulation of fæces must tend to induce a spasmodic action of the intestine, and this for a length of time repeated, may ultimately produce a more permanent state of contraction, increasing in proportion as the part becomes more deranged in its organization." 26, 27.

Mr. W. does not, by this, mean to exclude other contributary causes, as acrid substances taken into the stomach, a morbid state of the secretions, more especially the biliary, "which, by inducing an increased vascularity of the mucous membrane of the intestine, may prove an exciting cause of stricture." Those derangements in the colon, described by Dr. Parry and others, Mr. W. thinks, are often owing to strictures near the termination of that intestine, or in the rectum.

Many instances similar to that quoted from Dr. Lettsom, at page 29 of Mr. White's work, may be found in the pathological writings of Bonetus, Morgagni, and Lieutaud. We shall only take one case from the last of these three eminent authors, as a specimen.

" Quidam à triennio tympaniticus, ileo gravissimo corripitur: vomitus nimirum accedit stercorosus, cum singultu pertinacissimo. Brevi collabuntur vires; et intra triduum rebus humanis vale supremum dixit.

" Inter exenterationem reperitur, septem pollices supra rectum firma et callosa coarctatio intestini coli; veluti circumdatum filum id arctè constringeret. Stupenda erat dilatatio supra obicem, non solum coli, sed etiam aliorum intestinorum." Clar. De Haen. *Læsiones Abdominis, Lib. Primus. Observat.* 494.

*Symptomatology and Diagnosis.* As was before observed, the approach of intestinal stricture is slow and insidious. The symptoms more particularly indicating its existence in the rectum are, habitual costiveness, occasional uneasiness from a sense of fulness and flatulence in the course of the transverse arch, and especially of the sigmoid flexure of the colon, aggravated by certain kinds of food. This fulness may sometimes be felt externally, about the hollow of the left ilium. To this symptom are often added, acute pain in the course of the colon, with a sense of pressure when the fæces accumulate above the stricture; violent spasmodic contractions in different parts of the intestine; sense of tight girding round the body; all which symptoms are aggravated in proportion as the stricture is seated high up in the gut.

Sooner or later an uneasiness and difficulty are experienced in passing the fæces, which become gradually more scanty, smaller-figured than those that are natural, and often discharged with a squirt, and flatulence. When the stricture is high up in the rectum, the fæces will sometimes lodge below it, and appear of a natural size when discharged. This may lead to an erroneous opinion.

Pain of the back, about the sacrum, is a very common attendant on stricture in the rectum, and sometimes a primary symptom; the pain frequently shooting down the thighs, and in some instances to the soles of the feet. Hæmorrhage is also a frequent occurrence, as well as a mucous discharge. Mr. W. has found pain in the back part of the head a usual symptom of this disease.

In simple stricture, pain is only experienced on going to stool; in a scirrhus state of the rectum, the sufferings are not only greater at these times; but there is also, at other times, great pain about the sacrum, often shooting down the thighs, as well as a sense of burning heat and pain in the rectum. In this last deplorable disease, especially in its advanced stages, the fæces passed are generally in a liquid state, so that the disease may be confounded with a chronic dysenteric complaint. In strictures of the rectum, there is little emaciation or loss of strength until the disorder is far advanced; the countenance then becomes sallow; and, in some instances, the pulse is quick, with other hectic symptoms.

*Mode of Examination.* The first step to be taken, our author observes, (after the bowels have been freely opened) is to introduce the finger as high up the rectum as possible, desiring the patient to strain as at stool, by which means the stricture may sometimes be discovered. If a small bougie, however, be introduced, it is apt to hook upon a fold of intestine, and thus a stricture may be supposed to exist, when, in reality, there is none. If, therefore, on introducing the finger, neither stricture nor induration can be discovered (which Mr. White considers a favourable circumstance, as a scirrhous state of the rectum is generally within reach of the finger) a large-sized bougie is to be introduced, and pushed up as high as the colon, which will be readily done, if there be no obstruction in the passage; "because there may be a stricture at that part of the gut only, although we often meet with one two or three inches lower."

The situation of strictures in the alimentary canal is most commonly, Mr. White observes, about the termination of the colon; probably because the gut is naturally more exposed to pressure at its curvature, and at the projection of the sacrum, and consequently to accumulations of fæcal matters above this part.

"Strictures (says Dr. Willan) takes place in different situations; but they occur so frequently about the sigmoid flexure of the colon, near its termination in the rectum, that this part should be carefully examined in every case of total obstruction. The insertion of an unyielding tallow candle, though often practised, has been generally found painful and inefficacious. It is requisite for the purpose to employ a bougie thirteen inches long, and of a proportionate strength; which should also be directed with a nice hand, by a skilful surgeon."*

* *Reports, &c. p.* 135.

*Treatment.* Analogy would lead us to suppose that mechanical obstructions in the rectum would be relieved by mechanical means, in the same way as strictures in the urethra or œsophagus. Nevertheless, mechanical dilatation is not always applicable in any of these cases; and it is the object of Mr. White, in this section of his work, to shew where it is, and where it is not, to be employed in constrictions of the rectum.

Wiseman informs us, that in one case of intestinal stricture, he divided the part with an instrument successfully; in another, he used the actual cautery; but a subsequent pleurisy and dysentery proved fatal. Desault made use of a tent made of long lint, knotted and folded in the middle, dipped in cerate, and introduced into the rectum by means of a forked probe. This was removed twice a-day.

Dr. Darwin suggests, that the gut of an animal should be introduced, and then blown up with air, which is very nearly on the same principle as Mr. Arnott's dilator. Mr. Charles Bell recommended, some years ago, a sponge tent properly prepared, with small doses of calomel, to be purged off once or twice a week. Mr. Robert White suggests the probability of mercury being useful in a contracted state of the rectum, from its being serviceable in the schirro-contracted œsophagus; and Desault recommends the same treatment, particularly from his having frequently seen venereal symptoms connected with the diseased state of the rectum.

"Before we employ the means calculated to dilate the passage of the rectum, we should endeavour to ascertain not only the degree, but also the nature of the contraction, which is of great consequence; because it may happen that the diameter of the gut is less in a simple stricture than in a scirrhous state of the intestine; and yet, in the former instance, a dilatation of the passage may be effected, whilst in the latter case, a dilatation will not only be impracticable, but the introduction of a bougie under such a circumstance, may prove very injurious, by forming an additional source of irritation." 56.

After much experience and comparative trials, Mr. White finds that a tent, somewhat similar to Desault's, which, however he designates by the term "bougie," is preferable to the bougie, as commonly made, "not only from its being attended with much less inconvenience to the patient, but from the supposition that, by a continued gentle pressure, especially in a tuberculated state of the intestine, absorption would be more likely to be effected, than from employing the common bougie, which, in general, can only be retained a short time in the rectum." 56.

"From employing the common bougie in a few instances (where the stricture was as high up as the termination of the colon) the irritation was so great on introducing it, that I was led to make the tent somewhat stiffer, (but considerably less hard than the bougie) so as to be able to pass it as high as might be required." 57.

The bougie should be, at first, of such a size as to pass the stricture without considerable resistance, lest irritation and inflammation be excited. The size should also be increased very gradually till the parts become accustomed to the stimulus. There being always more or less of spasmodic action excited by the bougie, it should be introduced slowly and gently, waiting a little when it touches the stricture, before it is pushed through. At first it should not remain longer than half an hour in the rectum; if there be much irritation not so long. By degrees it may be allowed to remain eight or ten hours at a time, with little or no inconvenience to the patient. In general, it may be passed daily. From four or five to eight or ten weeks will elapse before the stricture admits a full-sized bougie; even then the instrument must be gradually left off. Mr. White has observed the natural action of the bowels to be generally much improved by the application of the bougie.

As auxiliaries, we may mention the hip-bath, and injections with extract of poppy. The former may be used for a few minutes before employing the bougie; and the anodyne injection after the bougie contributes to lessen the morbid irritability of the part.

In respect to the division of intestinal stricture by the knife, as practised by Wiseman and others, Mr. White thinks there can be no doubt of the occasional necessity for such an operation, when the bougie fails. The species of contraction noticed as the consequence of venereal infection, Mr. W. has found exasperated by the bougie, even when conjoined with a regular course of mercury. "In the tuberculated state, however, arising from a similar cause, the bougie will be found of great service." In scirrhus of the rectum the bougie would manifestly be improper; and it is on this account, Mr. White thinks, that some eminent men are averse to the employment of that instrument altogether in cases of contracted rectum.

In respect to the medical treatment, the first object is to regulate the bowels; laxative medicines being necessary, not only in the constipated state of the bowels, attendant on the early stage of the disease, but also in its more advanced progress, when a diarrhœa supervenes. Castor oil is to be preferred to any other medicine. Aloetic purges should be carefully avoided. Laxative glysters should be daily thrown up, if possible; for, by dissolving the fæces, their passage through the contracted part is greatly facilitated. Great attention, however, should be paid in throwing up glysters. They should be made of water gruel, with a table spoonful of castor oil, or sweet oil, or a little Castile soap dissolved in warm water. Sometimes warm water alone will be sufficient.

"When injections cannot be thrown up in the ordinary way, from the contracted state of the passage, a large hollow bougie may be fastened (instead of a common pipe) to a bladder, by which means they may be conveyed beyond the obstruction."

"I have prescribed mercury in some cases of scirrhus, and likewise in those of simple stricture; but I do not think it has ever been of any service in those cases of genuine scirrhus in which I have employed it. In simple stricture I am inclined to think more favourably of it. I have generally given the pil. hydrarg. combined with extr. conii. The latter, I think, tends to lessen the morbid irritability of the canal, whilst the former promotes a more regular discharge of bile. When the disorder is suspected to arise from a venereal cause, the exhibition of mercury is indispensibly necessary." P. 69.

In the advanced stage of scirrhus the sufferings of the patient are frequently so great as to render large and repeated doses of opium necessary. Extract of poppy and hyoscyamus are preferable, if they give sufficient ease, as they do not constipate. Would not the extract of strammonium or the lactucarium be useful in such cases? The local application of opium has not been productive of much effect in the hands of our author. The diet should consist of that kind of food which contains the greatest quantity of nourishment in the smallest compass. Jellies, sago, arrow root with milk, beef-tea, thin chocolate, fresh fish, eggs, either raw or lightly boiled, will be proper; with a sparing use of animal food. Fermented or vinous liquors are, of course, to be abstained from. Mr. J. M. Good, when he wrote his Nosology, had three patients under his care, who were *alleviated* by light liquid and aperient diet, without surgical assistance.

We may here take an opportunity of mentioning an instrument, called a *rectum syringe,* which we have found very useful in affections of the rectum and adjoining parts. It is sold at Evans and Co's, 11, Old Change, and probably other instrument-makers in London. By means of this, suppositories are conveniently applied, and often answer a better purpose than injections.*

* During the last stage of carcinomatous stricture, when the stomach will no longer retain food or medicine, and injections have no effect, or

In the course of the work, our author alludes to an Essay, containing an ingenious mode of introducing the rectum-bougie, communicated to him by Mr. J. M. Coley, of Bridgnorth, and read, about two years ago, at the Medico-Chirurgical Society. Mr. Coley was first induced to try this expedient in a case of stricture, which occurred in a gentleman, aged 73. All attempts to pass a bougie were found impracticable, in consequence of the oblique course of the stricture, the projection of the upper part of the sacrum, and a thickening of the intestine towards the bladder, of such extent, as to occasion most distressing strangury and hæmaturia. It was found impossible to introduce even a hollow tube, for the purpose of injecting an enema. Medicine was unavailing, and the most pressing danger was at hand. Dr. Du Gard, of Shrewsbury, was now consulted, who, being satisfied that it was impossible to convey a bougie into the stricture, advised a sponge-tent to be tried. This was also found to be of no avail. In short, all the ordinary resources of our art were attempted in vain, and the patient must certainly have perished, had it not occurred to Mr. Coley that the bougie might be defended from all intervening obstructions, by means of a tin canula, and thus prevented from bending upon itself; a circumstance which had hitherto foiled his endeavours. On trying this plan with a small canula, he readily introduced through the contracted part, such a bougie as would just enter an adult urethra; and by repeating the operation daily, and increasing the size of the bougie, he dilated the bowel in the course of two months, as far as was requisite. By these means, and the administration of pilula hydrargyri and aperients, the patient's health was restored, and his usual bulk regained. His stools became large and the action of the intestines regular. The tenesmus and copious discharge of mucus, together with the distressing symptoms noticed above, and that sudden jerk during the evacuation of the fæces, which is characteristic of stricture, subsided. He had an enlargement of the prostate gland, particularly of the right lateral lobe. The middle lobe did not appear to be diseased. The obstruction in the rectum had been observed about two years, and during the last forty years, he had been subject to frequent attacks of psoriasis and phlegmonous erysipelas. He had a

---

cannot be had recourse to, we have found that the constipation may be removed occasionally, and existence protracted by effecting the absorption of tincture of jalap, applied by means of friction on the abdominal parietes.

small polypous tumour below the stricture, and an external hæmorrhoid; neither of which produced inconvenience. During the cure, he retained the bougies eighteen hours daily, and walked about and rode on horseback, without displacing them. After a while, finding his strength and activity increase, and suffering some inconvenience from riding, he had the bougies introduced at four in the afternoon, and retained them until ten on the following morning, at which time he had regularly an intestinal evacuation. By this arrangement, his exercise and amusements had no interruption, The bougies were gradually discontinued. He had two relapses at the distance of about six months each time; but during the last year and a half he has been free from the disease. Through the whole cure, it was found impossible to make a bougie pass into the stricture, without the assistance of the canula. When the diameter of the canula was increased, to admit a larger bougie, Mr. Coley found that, by holding a lighted candle at the lower end, the orifice of the contracted part of the intestine could be readily distinguished. This we conceive must be a desirable object, inasmuch as it will assist us in ascertaining, in a situation *too distant for the finger to reach,* the appearance and nature of the disease under examination. The bougies he makes use of are similar to Mr. White's, excepting that they have a loop at the lower end, about three inches long, for the purpose of being secured to a T bandage. They are composed of lint rolled up, tied at the lower end with string, which forms the loop, and immersed in a composition of lead four parts, and wax one part; and, lastly, they are drawn through a wooden frame, having holes of various diameters. Great advantage, he observes, will be derived from making the points conical. His manner of applying them differs from our author's in this, that they are *wholly concealed within the rectum,* as will be presently described; which he considers a great improvement, as it enables the patient to walk about, or even ride on horseback, during the use of them. He advises them to remain in the bowel, if possible, all night; which, he thinks, has the effect of promoting the absorption of diseased structure, by long-continued pressure, as well as of resisting the tendency to contract. At the same time, he observes, that the discharge of the cerate, produced by the heat and moisture of the anus, is avoided; the cerate *not being melted by any portion of the intestine above the sphincter.*

As we consider this an important improvement in the treatment of this terrible disease, we feel disposed to afford it every possible publicity, and to recommend its adoption, when the bougie cannot otherwise be conveniently introduced, in every instance of simple permanent contraction in the ordinary situation; namely, from four to five inches, or higher up the bowel. No case should be abandoned until all rational means have been attempted; and should this measure possess no other advantages than that of enabling those to use it who might not have acquired the *tactus eruditus* of Mr. White, and that of exposing to view the orifice of the stricture and the state of the mucous membrane, it would, on these accounts, in our opinion, be deserving of record; and this, we believe, is the opinion of one of the first hospital surgeons in this metropolis.

The canulæ Mr. Coley employs are of three different sizes. They are cylinders, six inches long, turned down at each end, and below the upper extremity, perforated with holes in a row, within one-sixth of an inch from the margin. A small piece of wash-leather is to be bound round the edge through these holes, for the purpose of defending the inner coat of the intestine during its introduction. A piston of wood, rounded at the top, extending half an inch beyond the canula, and sliding freely within it, with a handle and stop at the bottom, should be had in readiness. The diameter of the smallest canula must be half an inch; of the next size, seven-eighths of an inch; and the largest, one inch and one-eighth. The pistons should be adapted to the different canulæ. The most favourable position for the patient is the same as for most other operations about the anus; the body should be bent forward, while the head and hands rest on the seat of a chair. The piston being placed with its corresponding canula, and the outside of them both being covered with butter, or fresh lard, they are to be gently introduced within the anus, the sphincter of which must also be anointed. The piston being withdrawn, the canula is to be pushed upwards, until it arrives at the stricture, which will be known by the resistance experienced. The patient should be desired to make one gentle effort, as though he were about to evacuate the intestine; during which act, the upper end of the canula may be readily adapted to the orifice. Should any difficulty happen in endeavouring to procure a view of the strictured part, the upper end of the canula must be gently moved in various directions; and, after the proper course has once been ascertained, future trouble may be avoided by adhering to the same position in the subsequent operations. By the pressure of the canula against the stricture, its lips are expanded, and the rugæ of the mucous membrane removed. The bougie should now be conveyed through the canula into the orifice of the stricture, when its progress will generally be suspended by a sudden spasm, which is involuntary, and imperceptible to the patient. During the continuance of this irritable state, no attempt must be made to carry forward the bougie. Having waited a few minutes, it may be pushed onward in a gentle manner; while the lower end of the canula is raised towards the apex of the os coccygis, and inclined to either side, according to the course of the stricture. By this combination of power so applied, as to make the long axis of the bougie approach towards a line with that of the stricture, all mechanical obstruction will be removed. The bougie should be kept steadily in its situation, and watching an opportunity, while the muscular fibres are relaxed and off their guard, it must be conveyed gently and steadily through the stricture. The length of the bougie need not exceed eight inches, which, being deposited in the situation that will presently be explained, will be equal in effect to one of ten inches employed in the usual manner; since it will be found to extend as far within the sigmoid flexure of the colon, as can ever be thought safe or desirable. When it has fairly passed beyond the obstruction, it should be propelled further, until its lower end has entered the canula to the distance of three or four inches, which may be effected by the assistance of the piston. While it is retained at this elevation by the piston, the canula must be drawn down to the stop, when the bougie will escape from the apparatus and lie across the pelvis, resting its lower end against the os coccygis or the levator ani, between that part and the tuberosity of the ischium. Lastly, the canula and piston are to be withdrawn.

Stricture is not so frequently met with in the sphincter ani as in situations higher up the alimentary canal. It has been accurately described, in a case published by Dr. Baillie, in the Medical Transactions, and noticed by our author, for the first time, in the present edition. We have sometimes found it accompanied with painful and troublesome ulcerations in the mucous membrane, occasioned by the passage of hardened fæces. The ulcers are mostly longitudinal, have a sloughy appearance, and are healed with difficulty. The best application is the nitrate of silver pointed like a pencil, or powdered, and laid on the bottom of the ulcerated surface. In one instance, the disposition to contract and ulcerate was entirely destroyed by a fistula, which rendered it necessary for us to divide the sphincter.

Before we quit this article, we cannot avoid expressing our astonishment, that an account of the plan of treatment laid down by our author is no where, we believe, to be found in that useful work, Mr. Cooper's Surgical Dictionary. We hope this omission will be rectified in another edition.

We have now laid before our readers a comprehensive view of the improvements that have been published in the treatment of the dreadful malady under consideration, and have condensed into one focus, we trust, much useful information; together with a pretty full analysis of the volume before us, more than half of which is occupied with a detail of valuable and interesting cases and dissections, which it is not possible for us to contract within any reasonable compass. When we reflect that the disease had almost entirely escaped the notice of the ancients, and that the learned professors of our own times used to be content with recommending liquid diet and pumps and syringes, with a view of relieving what they could not cure, we cannot withhold our praise from those who have so diligently pursued the subject, and discovered that contractions in the rectum may be successfully treated, even when situated at a much greater distance from the anus, than was formerly known to be within the reach of chirurgical aid.

"Homines nullâ re propiùs ad Deos accedunt, quàm hominibus *salutem* dando." CICERO.

Three editions of the work under review are sufficient proofs of public approbation, and we can only add our humble mite to the general suffrage. The economical and unostentatious manner in which the work is printed reflects credit on the author, and is deserving of imitation in times of universal distress like these. Medical authors would find their account in attending to this point.

